# Interoceptive posture awareness and accuracy: a novel photographic strategy towards making posture actionable

**DOI:** 10.3389/fnins.2024.1359594

**Published:** 2024-04-04

**Authors:** Steven P. Weiniger, Nathan D. Schilaty

**Affiliations:** ^1^College of Graduate Studies, University of Western States, Portland, OR, United States; ^2^BodyZone.com, Atlanta, GA, United States; ^3^Department of Neurosurgery and Brain Repair, University of South Florida, Tampa, FL, United States; ^4^Department of Medical Engineering, University of South Florida, Tampa, FL, United States; ^5^Center for Neuromusculoskeletal Research, University of South Florida, Tampa, FL, United States

**Keywords:** posture, interoception, photograph, image, energy regulation, sensorimotor, self-awareness, posture photogrammetry

## Abstract

Interoception, sometimes referred to as the ‘hidden sense,’ communicates the state of internal conditions for autonomic energy regulation and is important for human motor control as well as self-awareness. The insula, the cortex of interoception, integrates internal senses such as hunger, thirst and emotions. With input from the cerebellum and proprioceptive inputs, it creates a vast sensorimotor network essential for static posture and dynamic movement. With humans being bipedal to allow for improved mobility and energy utilization, greater neuromotor control is required to effectively stabilize and control the four postural zones of mass (i.e., head, torso, pelvis, and lower extremities) over the base of support. In a dynamic state, this neuromotor control that maintains verticality is critical, challenging energy management for somatic motor control as well as visceral and autonomic functions. In this perspective article, the authors promote a simple series of posture photographs to allow one to integrate more accurate alignment of their postural zones of mass with respect to the gravity line by correlating cortical interoception with cognitive feedback. Doing this focuses one on their body perception in space compared to the objective images. Strengthening interoceptive postural awareness can shift the net result of each zone of postural mass during day-to-day movement towards stronger posture biomechanics and can serve as an individualized strategy to optimize function, longevity, and rehabilitation.

## Introduction: life, energy and interoception

Interoception evolved around 180 million years ago as a crucial adaptation for warm-blooded mammals to optimize energy homeostasis while maintaining a constant internal temperature amidst fluctuating environmental conditions. An infolding buried under the brain’s Sylvian fissure – called the insula – is described by Craig as the cortical center of interoception (Craig, 2014). Referred to as a “hidden sense”, the new sensory capacity of interoception is, “the representation of the internal states of an organism, and includes the processes by which it senses, interprets, regulates, integrates, and modulates signals from within itself” (Chen et al., 2021). With a somatotopic homunculus that is tightly integrated with the homunculi of the sensory and motor cortices from which it embryologically descends, its “neural structures (a mix of viscerosensory, somatosensory, autonomic, peripheral, and central) cooperate to produce a real-time map of the homeostatic state of the human body” (Carvalho and Damasio, 2021).

While overall more energy efficient than quadrupeds, a biped’s alternating unipedal gait is inherently unstable, requiring tightly coupled spinal cord, brainstem, and cerebellar neuromusculoskeletal (NMS) reflexes to effectively manage energy and stay vertical. So as the only mammalian obligate bipeds, human biomechanics require greater neural control to manage energy, a key evolutionary development in which the insula plays a key role. As the cortical center of an interoceptive neuro-kinetic network (NKN), the insula coordinates cerebral and cerebellar cortical control of energy and muscle pattern synergies (Kumai et al., 2022).

All motion begins and ends with posture (Martin, 1977), and so biomechanically inefficient posture – and thus motion – is energetically costly. Energy efficient upright stance requires hierarchies and layers of mechanical and metabolic sensory feedback, paired with moment-by-moment predictive neural feed-forward motor control. Interoception senses energy use, and so perceives the energetic cost of inefficient biomechanics, an input perceived by the system as a stressor. In addition, the insula’s role in human cognition links proprioception and movement with interoception and emotion (Critchley and Garfinkel, 2017; Gao et al., 2019).

## Interoception in health and posture

**Figure 1:**
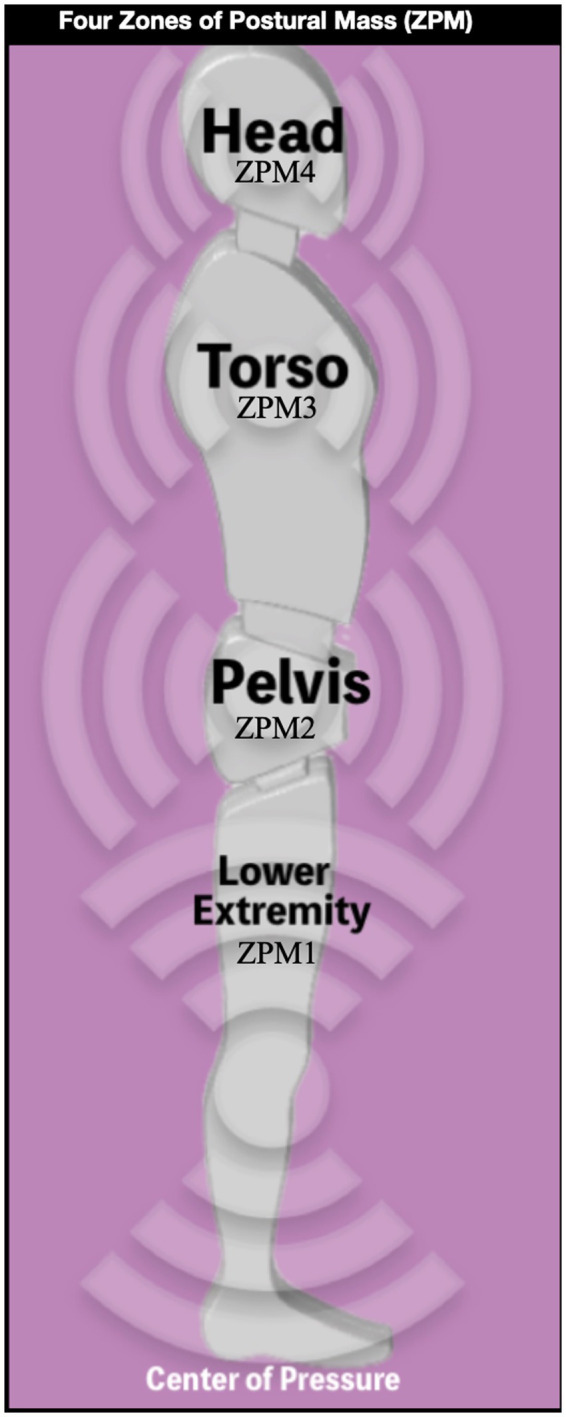
Four zones of postural mass (ZPM). Head, torso, and pelvis balance over the center of pressure of the lower extremity and feet. Moment by moment, at rest and in motion, these four ZPM are reflexively maintained in dynamic equilibrium via neural and interoceptive feedback with predictive control by the neurokinetic network (NKN). ZPM relative positions are limited by fascial, muscular and other soft tissue interconnections due to short-term functional compensations as well as longstanding structural adaptations. Image adapted with permission from BodyZone.com LLC 2020. All rights reserved. https://posturemonth.org/zones-of-postural-mass/.

Energy management is considered the brain’s most important biological task (Critchley et al., 2013; Barrett, 2020; Bruckmaier et al., 2020). As the cortical center of an NKN managing motion and energy, the insula weighs the perceived salience of sensory inputs against the actions of motor output, and so plays a significant role in human health and behavior (Tsakiris and De Preester, 2018). As evidenced by attempting to balance a manikin on two feet, even upright posture is an unstable structure, requiring accurate control to position 4 zones of postural mass (ZPM) towards vertical. The lower extremity (ZPM1) is the mobile base which supports the pelvis (ZPM2). Pelvic position drives the possible location and orientation of the torso (ZPM3). The torso drives the upper extremity in the possible locations for the center of control the head (ZPM1) to visually observe interactions between the person, their body, and the environment (Figure 1).

Bipedal gait requires alternating unipedal whole body balance and is more efficient when the ZPMs trend towards center (Hasegawa et al., 2017). To produce coordinated movement synergies for the whole organism, moment-by-moment non-conscious neural control of muscle patterns must interoceptively sense and predict positive and negative variance in position, velocity, and rates of acceleration, deceleration and momentum within and between adjacent ZPM, correcting for errors to continually adjust the changing relative contribution of forces upon the whole body, the collective four ZPMs.

The computational demands of bipedal neuromotor control is why though only 2% of body mass, the human brain consumes “20% of the energy expenditure” (Bruckmaier et al., 2020). “Half of small diameter sensory fibers from muscles signal metabolic energy expenditure” (Craig, 2014), inputting sensory information for energy utilization to the insula for the NKN to best manage mechanical energy to orient, move and control wobbling perturbations of these ZPM in highly variable bipedal gait and actions” (Papathanasiou et al., 2006). With respect to the precise control of energy use, efficient NMS function is a biomechanic mirror to the moment-by-moment biochemical balancing of reactive homeostasis and predictive allostasis, with NKN motor outputs adjusting each ZPM position in space, dependent on interoceptive monitoring of bodily energetic states. In humans, this bidirectional autonomic sensing and control for energy regulation underlies emotions, cognition, and motivations, acting as homeostatic influencers of behavior (Strigo and Craig, 2016).

## The interoceptive relevance of unipedal balance for bipedal posture

Bipedal locomotion is not a simplistic kinetic chain (KC), but a biomechanical web of motion and sensory information about what has just occurred, integrated with predictions of what may come next. In addition to static bipedal stance, the dynamic alternating unipedal balance of gait is a physical expression of each individual’s unconscious perceptions of their body’s physical location in space – their pattern of ZPM alignment. Combinations of synchronous and predictive contralateral motor sensors and effectors makes static posture and dynamic movement unique for everyone, and each motor task able to be executed in near infinite possibilities (Carriot et al., 2017).

Though unconscious, all volitional motions expend neural control and muscle energy, and global body movements that trend towards symmetric gait are more energy efficient (Helme et al., 2021). When postural masses asymmetrically diverge from the gravity line, energy use and stress asymmetrically propagate through the interoceptive NKN and biomechanical KCs. “Multisensory integration contributes to the perception of exteroceptive signals alongside an ever-present flow of interoceptive sense data” (Quigley et al., 2021). So, whether from pain, awkward activity, or habits of learned motor patterns, asymmetric mechanical stress requires the center of mass of adjacent ZPMs to shift away from alignment, and then back again for the upcoming step. Interoception senses the energy cost of inefficient posture, creating both a biomechanic as well as an interoceptive stressor, cumulatively contributing to allostatic load, towards systemic over-stress and allostatic overload.

Static posture has historically been simple to observe, measure, and compare. Unfortunately, this has led to labeling two states of static posture: Good or Bad. The complexity of human structure means the noise of individual variance drowns out the signal for assigning labels of good and bad. In other words, one person’s “good posture” may be another’s “bad posture” – confounding simplistic solutions. Despite common attribution of bad posture as a cause of back pain, the association is tenuous (Van Wesemael et al., 2024). In our modern tech-flexed society, unless one has a perfect body with no injury history and uncommonly above average activities of daily living (ADLs) including non-sedentary behavior and consistent exercise, “bad posture” or other labels often pathologize posture. Kendall and Kendall’s standard body mass alignment of “Tragus-Acromion-Trochanter-half an inch anterior to Malleolus” (Kendall et al., 1993) plumbline is outdated due to lifestyles and variances of ZPM mass, soft tissue function, joint angles and other biomechanic uniqueness. A more realistic goal is awareness of body symmetry and alignment coupled with efforts to manage biomechanic stress with more accurate postural awareness and control of symmetry in ADLs.

“Motion is lotion” is a maxim that applies to back pain as well as to healthy aging. Chiropractic, physical therapy, and other force-based intervention researchers overwhelming agree that for chronic back and neck pain, “manual therapy should be combined with exercise for nociceptive or neuropathic pain phenotypes” (Cook et al., 2023). However, all motions are not equal. Over time, asymmetry of static bipedal and unipedal balance propagates progressively weaker and more asymmetric patterns of static posture and dynamic motion, fostering inefficient energy use and greater biomechanical stress. Muscles strengthen with use, and “neurons that fire together, wire together,” summarizing the more complex neuroplastic concepts of Hebb’s law (Keysers and Gazzola, 2014).

However, globally, injury and/or pain on one lower extremity affects the biomechanic NMS and interoceptive NKN bilaterally, affecting asymmetries of motion bilaterally, thus further increasing risks of potential re-injury. Even 12 months after rehabilitation, pain-free athletes who have undergone anterior cruciate ligament reconstruction show asymmetric bilateral alterations in motor unit recruitment (Schilaty et al., 2022, 2023). Moreover, studies reveal that neuromuscular and proprioceptive training improve biomechanics and health outcomes (Nagai et al., 2023), supporting the speculation that pain generated asymmetries can propagate ongoing motion pattern asymmetries even after the injured soft tissue heals, contributing to development of chronic pain, and inhibiting exercise behaviors.

## Bio-behavioral relevance of weak posture

We contend that current technology behaviors, sedentary habits, and a weakening of one’s body awareness are interrelated with a societal degeneration of posture awareness and control. Lancet’s chronic back pain call to action guidelines advise exercise and a “positive health” concept, biopsychosocial approach (Buchbinder et al., 2018). Psychology and mindfulness behavioral studies define body awareness as “the subjective, phenomenological aspect of proprioception and interoception that enters conscious awareness which is modifiable by mental processes including attention, interpretation, appraisal, beliefs, memories, conditioning, attitudes and affect” (Mehling et al., 2011). Meta-analysis studies of body awareness and other mind–body interventions show changes in interoceptive accuracy can be an objective proxy for the intervention’s efficacy. Heartbeat tracking and detection as well as respiratory tracking, blood glucose accuracy, tactile sensitivity and joint position sense are proxies demonstrating somatic changes associated with emotional and psychologic stress (Treves et al., 2019).

Meta-analysis studies show reduced proprioception in low back pain sufferers compared to controls (Laird et al., 2014). Systematic review correlates forward head posture with impaired cervical proprioception, balance performance and reduced limits of stability (Han et al., 2016). Also, texting postures with the head flexed and the hands and shoulders rolled inward compress the torso, inhibiting full diaphragmatic excursion and respiratory vital capacity (Zafar et al., 2018).

Humans can choose to focus attention on subtleties, external and internal, to perceive and modulate interoceptive and homeostatic functions which are usually unconscious (Price et al., 2023), potentially affecting how people feel, think, and ultimately act (Nagai et al., 2023). A focused attention towards more accurate somatic and interoceptive perception of postural motor control can improve body awareness and motion pattern symmetry, contribute to improved rehabilitation success for NMS back and neck pain, strengthen balance and NKN for improved whole person health.

## A novel protocol for postural awareness

An interoceptive posture picture (IPP) is a series of four photographs as the person attempts to stand tall to capture their perceptions of the relative position of postural body masses with respect to true vertical. Two bipedal and two unipedal images with a standardized background grid allows visualization of how the individual physically positions their four ZPMs in space with respect to the centerline of the grid. Thus, the grid is a proxy for the gravity line, and the IPP a proxy for the interoceptive perception and expression of postural symmetry of the attempt to stand tall.

An IPP objectively benchmarks the individual’s perception of bipedal and unipedal postural balance, capturing the expressed sensorimotor and interoceptively influenced postural patterns. From a cognitive perspective, the attentional focus of having a picture taken while expressing the subjective feeling of “good posture” is a novel experience, and thus more likely to be encoded in memory. This allows comparison of future IPPs visually, as well as subjective correlation of the feeling of standing tall with their cognitive, proprioceptive and interoceptive perceptions of postural symmetries.

Like blood pressure being a “snapshot” of arterial health motivating better health choices, an IPP can make a clinician’s communication more relevant by facilitating a two-way communication highway of physical structure and behavioral function. Focusing attention on a single body mass – head, torso, pelvis, or lower extremity – allows more precise execution of cueing instructions for rehabilitation or other exercise. More accurate awareness of postural patterns can improve the ability to control those patterns, impacting interoception, proprioception and motor control for improved biomechanic efficiency, and towards reducing injury.

**Figure 2:**
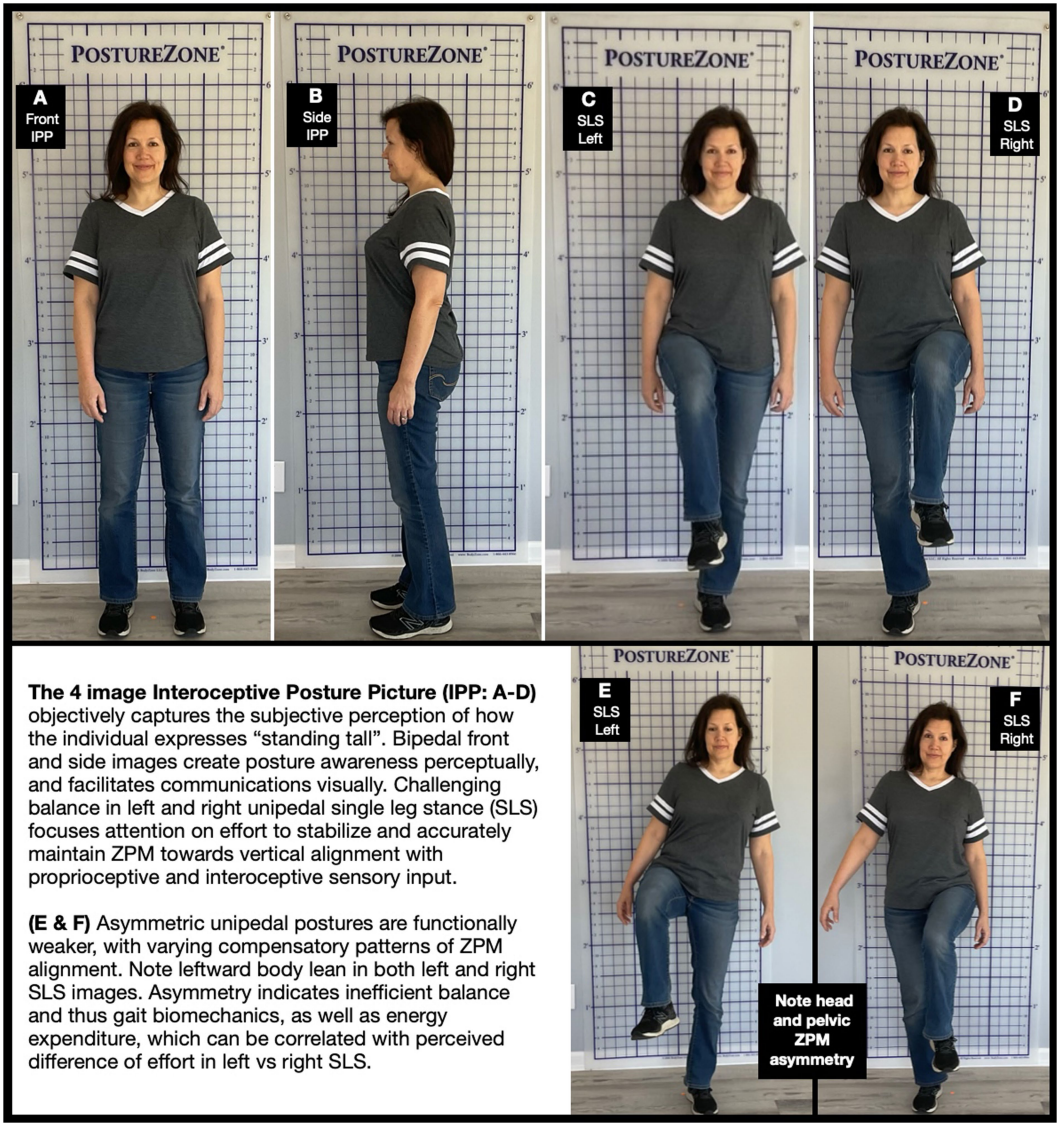
Interoceptive posture picture (IPP) **(A–D)** + Asymmetric unipedal postural pattern **(E,F)**. An IPP visually and perceptually communicates posture awareness with unipedal and bipedal posture images using a standardized grid to compare symmetry and ZPM deviations from centerline. Specifically attempting to “stand tall” focuses attention on feeling interoceptive and proprioceptive sensations. **(A,B)** Frontal and side images to correlate cognitive awareness of posture with subjective perception of standing tall. **(C,D)** Unipedal single leg stance (SLS) challenges balance to document asymmetric patterns of attempting to stand tall. Expending effort to perform focuses attention on proprioceptive and interoceptive sensations, allowing comparison with cognitive perceptions of ability to control balance and symmetry. **(E,F)** Asymmetric Left-Right SLS: Note body lean to left with both standing legs. Unequal ZPM deviations indicates inefficient balance biomechanics and energy expenditure. Asymmetry can be correlated with perceived difference in left vs right effort. Image used with permission from cesoup.com. https://cesoup.com/posture-rehab/.

The protocol for an IPP begins with bipedal images. For the frontal view, a person is instructed to stand tall with relaxed posture, and center to a dot on the floor that is aligned with the grid centerline. However, experience suggests that most do not express their usual resting stance, but rather their cognitive ideal of how their body should stand. Verbal cues such as “take 3 steps in place” can shift awareness away from common exaggerations of rigid postures for a more unthinking, proprioceptive and interoceptive perception of standing tall. In Figure 2A, the body is bisected by the grid centerline, and lateral aspects of pelvis and torso similarly displaced from the edge of the grid. Next, instruct the person to face right for the lateral bipedal image (Figure 2B). This allows visualization of forward head posture with respect to torso or centerline, as well as relative lateral ZPM positions with respect to the grid edges.

The second set of IPP images are unipedal, integrating a widely used Single Leg Stance (SLS) test. SLS images allow comparison of relative strength and accuracy of global left versus right side weight bearing strategies for postural balance. Adding the “stand tall” instruction in addition to a grid objectively captures relative asymmetry in patterns of ZPM alignment, as well as how that expressed posture is subjectively perceived.

For each side, the subject is instructed to face forward, stand tall, and to the best of their ability raise the thigh up towards level. Frontal SLS images (Figure 2C SLS-L and Figure 2D SLS-R, respectively) show left and right leg weight-bearing unipedal balance with near symmetry of the, respectively, lifted legs, with pelvis, torso and head aligned towards the vertical gridlines close to the supporting foot.

Grossly asymmetric and incongruent patterns of ZPM alignment in SLS indicate asymmetric motor control patterns, and thus inefficient NKN management of kinetic energy. The lifted leg deviating from center and lateral body lean is evident in Figure 2E. In Figure 2F, the contralateral leg midline crossover of the lifted leg indicates compensatory effort, pain or mechanical stress. In both these images, PZM alignment is visibly impacted. However, the leftward global body deviation shown in both Figures 2E,F demonstrates very different patterns of stabilization, and thus energy expenditure.

## Fostering interoceptive posture awareness and accuracy

Humans can choose to focus attention on subtleties, external and internal, to perceive and modulate interoceptive and homeostatic functions which are usually outside of awareness (Price et al., 2023). Most can perceive and control breathing, and with mindfulness training, often even heartbeat (Schillings et al., 2022), and so affect how they feel, think, and ultimately act. Initially, integrating interoceptive attentional focus and cognitive awareness of the body with an objective picture allows people to become conscious of an interoceptive blind spot: “What does my posture feel like?” Over time, systematically focusing cognitive attention on internal feelings of the somatic motor expression of “standing tall” creates awareness and increases the salience of incoming interoception (Farb et al., 2022; Schmitt and Schoen, 2022; Rineau et al., 2023).

Global as well as ZPM structural asymmetry can be a benchmark for asymmetric interoceptive NKN management of kinetic energy. Benchmarked and tracked, an IPP can be a visible proxy screening for the interaction of interoceptive postural awareness and accuracy (IPA&A), and so can contribute to body–mind approaches to strengthen self-awareness and self-efficacy.

An IPP provides a non-pathologizing experience and biomechanical reference of an individual’s unique static standing posture against a standardized background, potentially strengthening the subjective IPA&A of how they perceive their body in space. Mindfully and cognitively focusing to proprioceptively align ZPMs towards objective vertical requires multi-sensory integration. This volitional body-mind connection correlates visual, proprioceptive and interoceptive inputs regarding the positional motor control of ZPM symmetry, and so is a screen for their somatic expression of IPA&A.

Balancing on one leg requires a lateral pelvic shift, and in reaction the torso and head must shift as well. In efficient gait, these ZPM shifts are nearly symmetrical, and postural patterns of static bipedal and unipedal balance are largely congruent with those observed in gait. Greater structural asymmetry is a significant observation in low back pain (Al-Eisa et al., 2004), chronic neck pain (Kirmizi et al., 2019), and other NMS conditions, although ascribing the cause of asymmetry requires further clinical investigation. Over time, people become accustomed to asymmetries, impacting proprioceptive and interoceptive accuracy, in turn affecting fluidity in gait and effectively coupled coordination in activities of daily living.

Screenings with an IPP can benchmark IPA&A and build a greater multi-sensory awareness of standing tall towards a truer, more accurate biomechanic symmetry. Focusing IPA&A on subtle body mass alignment asymmetries is a strategy to develop habits to shift the individual’s ZPMs towards gravity line symmetry in ADLs. Focused attention towards more accurate somatic and interoceptive perception of postural motor control can improve body awareness and motion pattern symmetry, over time increasing their perceived relevance of improving postural habits and behaviors.

Clinically, IPP observed shifts in ZPM alignment can reflect the effectiveness of goal directed therapeutic interventions like progressive graded exercise protocols – objectively, as well as proprioceptively for the individual. Shifting learned patterns of motion towards objective biomechanical accuracy can improve rehabilitative success for NMS back and neck pain, as well as strengthen balance. Since interoception plays a key role in emotions, training stronger IPA&A of “standing tall” can be a powerful biopsychosocial tool to encourage self-efficacy, thus improving overall whole person health as well.

## Call to action

We contend that IPA&A is a biomechanic and interoceptive modulator of behavior which impacts NMS as well as whole person health as defined by the National Institutes of Health (NCCIH, 2021). Especially with forward head posture and other modern technology postures, an IPP is a screening tool and benchmark to objectively document patterns of ZPM alignment, and subjectively correlate proprioceptive and interoceptive perception towards visual reality. Building IPA&A with IPP images is a “minimal cost” strategy that can impact behaviors and improve global patterns of movement.

Genetically and behaviorally, everyone’s injuries, habits, and ADL behaviors are uniquely vulnerable (and robust) with respect to structural, environmental, and emotional stressors. Energetic tradeoffs between biochemical, biomechanical, and emotional strengths and weaknesses (i.e., habits, fears, and desires) interoceptively guide learned and reflexive patterns of perceptions and feelings to mold behavior.

Focusing awareness on accurately perceiving one’s posture and strengthening balance control is a strategy to optimize rehabilitation for that individual’s body (Cramer et al., 2018; Schilaty et al., 2023) as well as attitude and self-image. Benchmarking an individual’s initial perception of their posture by accurately focusing attention on discrete ZPMs with standardized images provides a more granular interoceptive baseline from which to retrain control of the postural NKN towards greater accurate gravity-line symmetry. Over time, conscious attention and effort to usually unconscious interoceptive signals can improve biomechanics and energy management, reshaping the NKN, reducing biomechanical stress of ADLs and improving the effectiveness of exercising.

Future studies should address correlations between interoception and posture, including strategies to foster more accurate sensorimotor control via strengthening interoceptive awareness, sensibility, and accuracy. Neuromusculoskeletal rehabilitation and performance programs should investigate integrating interoceptive attentional focus with IPP and ZPM alignment protocols.

Multidisciplinary biopsychosocial investigation should focus on creating somatic self-efficacy towards positive ADL behavioral modification. For self-care, promoting IPA&A towards standing and living taller during day-to-day movement can enhance energy optimization and thus biomechanical and even emotional resilience against everyday stressors as part of an individualized strategy to optimize function for successful aging and a healthier life.

## Data availability statement

The original contributions presented in the study are included in the article/supplementary material, further inquiries can be directed to the corresponding author.

## Author contributions

SW: Conceptualization, Resources, Writing – original draft, Writing – review & editing. NS: Conceptualization, Funding acquisition, Writing – review & editing, Supervision.
